# Deficient Mismatch Repair and Microsatellite Instability in Solid Tumors

**DOI:** 10.3390/ijms26094394

**Published:** 2025-05-06

**Authors:** Joy A. Awosika, James L. Gulley, Danielle M. Pastor

**Affiliations:** 1 Gastrointestinal Malignancies Section, Thoracic & GI Malignancies Branch, Center for Cancer Research, National Cancer Institute, National Institutes of Health, Bethesda, MD 20892, USA; 2 Center for Immuno-Oncology, Center for Cancer Research, National Cancer Institute, National Institutes of Health, Bethesda, MD 20892, USA

**Keywords:** microsatellite instability, deficient mismatch repair, tumor mutational burden, neoantigens, Lynch syndrome, hereditary non-polyposis colon cancer, tumor immune microenvironment, immune checkpoint inhibition, immunotherapy

## Abstract

The integrity of the genome is maintained by mismatch repair (MMR) proteins that recognize and repair base mismatches and insertion/deletion errors generated during DNA replication and recombination. A defective MMR system results in genome-wide instability and the progressive accumulation of mutations. Tumors exhibiting deficient MMR (dMMR) and/or high levels of microsatellite instability (termed “microsatellite instability high”, or MSI-H) have been shown to possess fundamental differences in clinical, pathological, and molecular characteristics, distinguishing them from their “microsatellite stable” (MSS) counterparts. Molecularly, they are defined by a high mutational burden, genetic instability, and a distinctive immune profile. Their distinct genetic and immunological profiles have made dMMR/MSI-H tumors particularly amenable to treatment with immune checkpoint inhibitors (ICIs). The ongoing development of biomarker-driven therapies and the evaluation of novel combinations of immune-based therapies, with or without the use of conventional cytotoxic treatment regimens, continue to refine treatment strategies with the goals of maximizing therapeutic efficacy and survival outcomes in this distinct patient population. Moreover, the resultant knowledge of the mechanisms by which these features are suspected to render these tumors more responsive, overall, to immunotherapy may provide information regarding the potential optimization of this therapeutic approach in tumors with proficient MMR (pMMR)/MSS tumors.

## 1. Introduction

**Figure 1:**
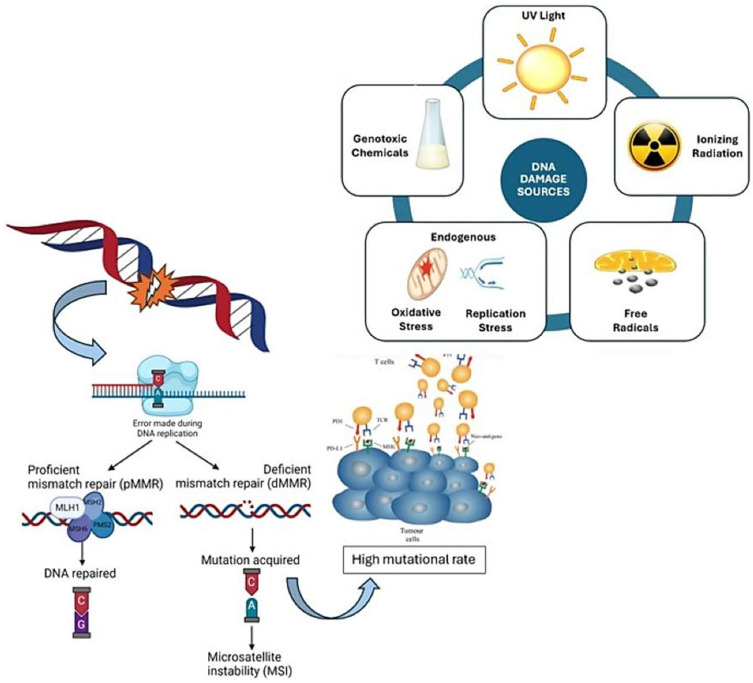
Mechanism of microsatellite instability and mutator phenotype. Schematic illustration of common sources of DNA damage that contribute to microsatellite instability (MSI), contrasting proficient and deficient DNA mismatch repair (MMR), the latter a prominent feature of MSI-high (MSI-H) tumors. This figure highlights how the accumulated replication errors that are characteristic of MSI-H tumors result in high tumor mutational burden, neoantigen generation, and the development of an immune-rich tumor microenvironment characterized by an increased population of tumor-infiltrating lymphocytes and immune checkpoint upregulation. Copyright: image elements adapted from iStock (credit: ttsz) and BioRender version 201 (credit: Ryan Denu), with modifications by the authors.

The oncogenic descriptions of dMMR and MSI-H tumors are delineations that have become pivotal in the classification of solid tumors. Microsatellite sequences are abundant throughout the genome and are unique and uniform in length in every tissue in each person [1]. These sequences demonstrate high polymorphism and are characterized as repetitive segments of DNA consisting of short motifs of nucleotides that are repeated [2]. DNA damage accumulation in cells is attributed to either exposure to external insults such as chemicals, ultraviolet light, and endogenous reactive metabolites, or errors occurring during normal DNA replication [3,4]. If left unrepaired, DNA damage generates mutations that may lead to tumorigenesis. Cells are equipped to respond to DNA damage through various DNA repair pathways. The process of MMR is regulated through such a pathway, activated via the recognition of base mismatches that have emerged during replication, and insertion–deletion loops (IDLs) within repetitive DNA sequences that have resulted from strand slippage events [4,5]. Microsatellite instability arises from defects in this DNA MMR system, a process that is critical in the preservation of genomic stability (Figure 1).

The criteria for successful MMR involves the initial recognition of base pair mismatches and IDLs followed by the direction of repair machinery to the newly synthesized DNA strand, which carries the erroneous genetic information [5]. Five MutS homologues (MSH) have been described in human cells and comprise the MMR proteins. The heterodimers Mutator Sα, consisting of MSH2 and MSH6, and Mutator Sβ, consisting of MSH2 and MSH3, sense the deletion, insertion, and mismatch site on the DNA strand. Afterward, the Mutator L-related complexes (MLH1/PMS2 or MLH1/MLH3, with the former complex playing the predominant role) cleave the lesion site [5,6]. These events ultimately result in a reduction in replication-associated errors; thus, an increase in the spontaneous mutation rate is evident when this pathway is aberrant due to the loss of one or more of the MMR proteins [3]. The resultant mutational phenotype leads to cancer development of either a somatic or germline origin. The high mutational burden produces neoantigens, making them susceptible to recognition and apoptosis via the adaptive immune system [7]. This phenomenon is commonly referred to as the tumor mutational burden (TMB). It is important to note that while the terms MSI-H and dMMR are often used interchangeably to suggest a fundamental defective cell state of genomic instability resulting from an aberration in DNA MMR, the TMB reflects a tumor characteristic that is distinct from dMMR/MSI-H. Both high-TMB and dMMR/MSI-H tumors have positive correlations with tumor immunogenicity and responses to immunotherapy; however, TMB is a quantitative feature that describes the number of mutations in the coding region of the genome (exome) of the tumor cells [7]. Tumor mutational burden is reported as the number of mutations present in a megabase of the genomic region as determined by whole-exome sequencing or large-scale next-generation sequencing; a higher TMB generally correlates with a greater probability of tumor neoantigen production and more robust T cell responses, potentially contributing to the improved response to immunotherapy-based treatments [7,8,9,10].

The status of MSI and MMR has both predictive and prognostic implications in most solid tumors. Testing for dMMR is achieved through the determination of the presence or absence of MMR proteins via the immunohistochemistry (IHC) staining of tissue samples, while the MSI status is assessed via polymerase chain reaction (PCR) amplification of a set of nucleotide repeat markers. These markers are then compared between tumor and normal DNA to detect somatic changes [11]. The MSI-H phenotype is well-described in colorectal, endometrial, gastric, and prostate cancers, and recognition in other solid tumors has been heightened due to increased frequency in and familiarity with testing for dMMR/MSI-H. This review aims to provide a comprehensive analysis of MSI-H solid tumors, covering the associated molecular basis, clinical characteristics, diagnostic methods, prognostic implications, and treatment strategies.

## 2. The Landscape of Deficient MMR and MSI in Solid Tumors

### 2.1. Colorectal Cancers

Several molecular pathways can lead to colorectal cancer (CRC) with MSI. Hereditary syndromes known to confer an increased risk of CRC development in individuals carrying pathognomonic pathogenic variants (with these resultant tumors either exhibiting MSI or features similar to those occurring in MSI-CRCs) include Lynch syndrome (LS), MUTYH-associated polyposis (MAP), and polymerase proofreading-associated polyposis (PPAP). While the development of CRC in the setting of hereditary syndromes typically results from the sequelae of germline mutations in genes associated with DNA damage repair, the more common non-familial form of MSI-H CRC is due to the epigenetic inactivation of *MLH1* occurring in a background of hypermethylation [12].

#### 2.1.1. Lynch Syndrome

Colon and rectal cancers exhibiting MSI may develop in the setting of an inherited syndrome or via a non-familial or non-hereditary, or “sporadic”, process. Formerly termed hereditary nonpolyposis coli or hereditary nonpolyposis colon cancer (HNPCC), LS is an inherited disorder that is associated with an increased risk of the development of various types of malignancies, of which colorectal and endometrial are the most common. In recent years, the use of the term “Lynch syndrome” has been more widely accepted as more precise nomenclature when referring to the syndrome that results in the presence of a germline mutation in DNA mismatch repair genes; in contrast, HNPCC is a clinical definition that refers to the satisfaction of criteria (e.g., Amsterdam criteria; Bethesda guidelines) related to personal and family history meant to identify those individuals who should undergo genetic testing [12,13,14,15,16]. Moreover, the designation of HNPCC may also refer to cases of familial CRC that exhibit MSI and other features of LS but lack germline mutations involving MMR genes (“Lynch-like” syndromes) or appear to be familial but are MSS and lack germline mutations (“familial colorectal cancer type X”) [12,14,17,18,19,20,21].

As one of the most common hereditary cancer syndromes, LS gives rise to 3–5% of MSI-H CRCs. Pathogenic germline mutations in the DNA MMR genes *MLH1*, *MSH2*, *MSH6*, and *PMS2* leading to an MMR functional deficiency are defining features of LS [22]. Although the inheritance pattern is autosomal dominant, the pathology ultimately develops from a second event involving the unaffected allele through which the resultant DNA repair dysfunction causes an increase in the frequency of somatic mutations in the cell line and an acceleration in malignant degeneration, translating to a higher risk of malignant transformation within a short timeframe pattern [15,23,24,25,26]. The cumulative lifetime risk of developing CRC in this patient population can be as high as 60–80% without surveillance and therapeutic intervention, although penetrance depends on the involved gene [27]. Mutations in the *MLH1* and *MSH2* genes have a greater effect on DNA repair and more frequently contribute to the manifestation of the typical CRCs found in individuals with LS, while mutations in *PMS2* and *MSH6* have distinct risks and patterns of development of intestinal and extracolonic malignant processes [28,29,30,31,32]. Germline deletions occurring in the last few exons of the epithelial cellular adhesion molecule (*EPCAM)* gene are also associated with LS, as such mutations lead to the epigenetic silencing of the *MSH2* gene, which is located downstream of *EPCAM* [33,34].

In more recent years, the term “Lynch syndrome” has been increasingly adopted in both the clinical and molecular platforms as more precise nomenclature to highlight the fundamental underlying genetic defect placing affected patients at risk for not only CRC, but for the development of multiple extracolonic neoplasms, which include, but are not limited to, endometrial, gastric, urinary tract, ovarian, and biliary tract malignancies [31,32]. This recognition of varying risk for the development of several types of neoplasms has led to the creation of clinical practice guidelines by organizations such as the American Society of Clinical Oncology, the European Society for Medical Oncology, the American College of Gastroenterology, and the U.S. Multi-Society Task Force, amongst others, to facilitate the early detection of these cancers and, thus, allow for potential curative treatment options [35,36,37,38,39].

#### 2.1.2. MUTYH-Associated Polyposis

MUTYH-associated polyposis is a unique syndrome characterized by a lifetime risk of CRC of up to 80–100% that, when CRC develops, shares characteristics with both MSI-H and CRCs exhibiting chromosomal instability (CIN) [40]. Inheritance occurs via an autosomal recessive pattern, caused by the inactivation of MUTYH, a base excision repair (BER) glycosylase involved in the repair of DNA damage induced by guanine nucleotide oxidation. The *MUTYH* mutation-mediated disruption of BER machinery results in the accumulation of G > T somatic mutations, which has been proposed to evoke anti-tumor immune responses similar to those evident in MSI-H tumors [41]. The overall mutation rate in MAP-associated carcinomas has been estimated to be approximately twofold higher than the rate estimated in MSS carcinomas, but still lower than the rate in MSI CRCs (which is almost tenfold higher than in MSS carcinomas) [42]. The higher somatic mutation load demonstrated in MAP tumors compared to MSS tumors is suspected to cause a more activated immune system, one that may be comparable to that which is evident in LS patients and in those individuals with sporadic dMMR CRCs. Like MSI-H tumors, MAP-associated CRCs are prone to lose human leukocyte antigen (HLA) class I expression, the result of which may promote the evasion of tumor surveillance, thus allowing these tumors to avoid recognition and destruction [43,44]. However, although HLA class I expression loss is a feature shared among sporadic MSI-H CRCs, LS-associated CRCs, and MAP CRCs, the mechanisms responsible for such expression loss is quite distinct between sporadic and hereditary populations [45,46]. In sporadic MSI-H CRCs, HLA class I expression loss appears to be the result of genetic defects in antigen-processing machinery components. In contrast, MAP-associated CRCs often fail to express beta-2-microglobulin, which is critical for cell surface HLA class I antigen expression [43,45]. This phenomenon is similarly seen in LS-associated CRCs. Given the higher mutation rate and potential to evoke an immune response, it is reasonable to hypothesize that MAP-associated CRCs may be sensitive to immunotherapeutic strategies, as are a proportion of MSI-H CRCs [47]. These strategies could possibly include vaccines and ICIs, trials of which are currently ongoing for use in LS patients in the prevention setting (NCT05419011, NCT04711434, NCT05078866, and NCT01885702).

Estimated to account for approximately 0.7–1% of all CRCs, these tumors that arise in individuals with MAP harbor an unusual type of CIN that is characterized by a loss of heterozygosity (LOH), without alterations in the chromosomal copy number (a phenomenon known as copy-neutral LOH), distinguishing them from other CRCs with CIN [48,49]. Though MAP-associated CRCs largely tend to be MSS, paradoxically, they are commonly right-sided/proximal in location, are often mucinous, and have an increased presence of tumor-infiltrating lymphocytes (TILs), characteristics that are known to be associated with LS-CRCs [48,50,51].

Regarding behavior, MAP CRCs have been shown to metastasize to a lesser degree than sporadic CRCs, but more so than LS-associated CRCs. Although predominantly MSS, reports have been published that suggest that a small number of MAP CRCs may exhibit MSI and/or dMMR, but the importance of these findings as they relate to the response of tumors to immunotherapeutic agents is unclear [52].

#### 2.1.3. Polymerase Proofreading-Associated Polyposis

Polymerase proofreading-associated polyposis is another hereditary CRC syndrome that bears overlapping features with MSI-H CRCs. It is an autosomal dominantly inherited syndrome caused by pathogenic germline exonuclease domain variants of *POLE* and *POLD1* genes, which encode the catalytic subunits of the DNA polymerases epsilon and delta that play important roles in DNA replication and proofreading. Colorectal cancers arising in the setting of PPAP account for only 0.1–0.25% of all CRCs, are more commonly proximal in location, tend to occur at a young age, are diagnosed at earlier stages, and have been shown to be associated with good prognosis [53,54]. Like MSI-H tumors, *POLE*-mutated tumors are considered to express a strongly mutated phenotype [55]. While reports have suggested that somatic *POLE* mutations may serve as potential molecular markers for predicting the efficacy of immunotherapy, data regarding this association in CRCs that harbor germline pathogenic variants are extremely sparse [53,56,57,58,59].

#### 2.1.4. Sporadic Colorectal Cancers

While CRCs arising in the setting of hereditary syndromes represent the smaller constituent of CRCs expressing MSI, the remaining 10–12% of MSI CRCs are comprised by those developing in a sporadic pattern in individuals lacking any demonstrable familial predisposition. This phenomenon is largely due to the epigenetic inactivation of *MLH1* occurring in a background of hypermethylation [60,61]. The promoter methylation of *MLH1* resulting in the gene’s functional deactivation may be caused by somatic mutations in the *BRAF* gene [28]. The presence of the V600E mutation in the *BRAF* gene, in particular, suggests a non-hereditary etiology, as the *BRAF* mutation is a rare occurrence in the presence of MMR gene germline mutation. Conversely, *BRAF* mutations are present in approximately 50% of MSI CRCs arising in a sporadic setting [62]. Thus, the coexistence of a somatic *BRAF* mutation and tumor dMMR/MSI-H status not only suggests a sporadic origin but may also be a marker for prognostic risk stratification in metastatic CRC, as *BRAF*-mutated tumors exhibiting MSI-H have been associated with poorer survival in the metastatic setting [63,64,65]. This is in contrast to the improved outcomes observed with early-stage MSI-H CRC, overall; it is possible that in advanced or late-stage CRC exhibiting MSI-H, *BRAF* mutations may be the driving force behind these documented poor outcomes [63,66].

The underlying type of genetic instability and the presence of DNA methylation in sporadic MSI CRCs suggest that this type of CRC originates from serrated polyps and is delineated by the CpG island methylator phenotype (CIMP) high status, methylation of *MLHI*, *BRAF* mutation, and MSI-H and is chromosomally stable. Based on a proposed molecular classification for CRC that considers morphological and molecular parameters, sporadic MSI CRCs share features of those tumors comprising “group 1” (CIMP-high/MSI-H/BRAF mutation) [23]. While the final pathway of both sporadic and hereditary MSI-H CRC converges with respect to an MMR deficiency, there are notable differences in age, gender, IHC profiles, molecular changes, and histopathological features between MSI CRCs that arise in individuals harboring pathognomonic germline variants versus those that develop in a non-familial sporadic pattern (Table 1) [67,68].

### 2.2. Endometrial Cancer

Among its subtypes, MSI-H endometrial cancer (EC) has gained attention due to its distinct molecular and clinical features. A MSI-H status is observed in approximately 20–30% of ECs and is associated with specific genetic alterations and clinical outcomes. The MSI-H, or hypermutated phenotype, is one of the four categories of EC distinguished by genomic characterization in The Cancer Genome Atlas (TCGA) [11,69]. A significant proportion of MSI-H ECs are linked to LS; moreover, the data suggest that a significant proportion of women with LS will present with EC as their initial cancer diagnosis [70]. Universal tumor testing for dMMR/MSI is recommended for EC at diagnosis and incorporated in several guidelines, such as the Society of Gynecologic Oncology and the National Comprehensive Cancer Network [71,72,73]. Unlike CRC, evaluating EPCAM loss and *BRAF* mutation has not been as relevant for patients with EC. While *EPCAM*-associated LS is often linked with CRC, it is much less common for individuals with this syndrome to develop EC [33,74]. Microsatellite instability-high ECs are often associated with specific clinical and pathological features. These characteristics involve an endometroid histology, low tumor stage, and histopathological features such as lymphocytic infiltration and tumor heterogeneity. The propensity for extrauterine metastases is higher in dMMR EC due to frequently observed lymphovascular invasion (LVI) and deep myometrial involvement [75]. The prognostic implications in MSI-H EC are not well defined, with several studies demonstrating mixed observations [74,76,77,78]. Some studies suggest that MSI-H ECs have a better prognosis due to increased immunogenicity, which enhances the anti-tumor immune response; however, other studies indicate no significant difference in survival rates compared to MSS tumors [77,78]. The molecular mechanisms of MSI-H ECs predominantly involve epigenetic MLH1 promoter hypermethylation which is observed in 70–75% of cases followed by somatic (15–20%) and germline (5–10%) mutations in *MLH1*, *MSH2*, *MSH6*, and *PMS2* [79,80,81]. *hMLH1* promoter hypermethylation-associated EC demonstrates distinct clinicopathological features such as older age, obesity, and more advanced stages with higher rates of LVI. Early-stage EC with *MLH1* promoter hypermethylation has been associated with worse clinical outcomes, demonstrated by shorter progression-free survival (PFS). Distinct molecular and immune profiles associated with this subgroup demonstrate an enrichment of *JAK1* mutations and lower TMB and TIL scores when compared with germline and somatic MMR gene-mutated EC [79].

### 2.3. Gastric Cancer

Approximately 15–30% of sporadic gastric cancers (GCs) exhibit high levels of MSI [82]. Gastric cancers that are MSI-H constitute one of the four molecular subtypes of gastric and gastroesophageal junction tumors according to the TCGA [83]. Gastric cancers exhibiting MSI-H are often located in the antrum or body of the stomach, typically present as intestinal-type adenocarcinomas, and are characterized by high levels of lymphocyte infiltration. These MSI-H GCs tend to occur in older patients and are more common in females. Favorable prognosis is observed, with a lower incidence of lymph node metastasis, lower pTNM stage, and better survival rate [84,85,86]. These improved rates may be due to a higher neoantigen load of dMMR/MSI-H tumors stimulating an antitumor immune response, hence reducing the likelihood of metastases [87]. Defects of the MMR system and MSI play an important role in the early stages of gastric carcinogenesis. Kim et al. observed that, in resected specimens of adenomas and carcinomas, 21% of gastric adenomas exhibited MSI-H; furthermore, 62% of adenomas exhibiting some level of MSI were associated with carcinoma, whereas 40% of MSS adenomas were associated with carcinoma [88]. Their observations also suggest that GCs arising from adenoma precursor lesions are frequently associated with a dMMR mechanism. This presence of MSI was maintained throughout the process of malignant transformation [85,88]. Defective MMR and a loss of hMLH1 or hMSH2 expression underly the MSI phenotype in MSI-H gastric tumors. Several studies have shown that the MSI-H sporadic GC is mainly due to the inactivation of *hMLH1* by the epigenetic silencing of the gene [85,89,90], while most tumors with a loss of hMSH2 expression have germline *hMSH2* mutations [60,91]. In addition, MSI-H GCs demonstrate distinct genotypes with an increased mutational frequency in tumor suppressor genes that have been shown to be critical targets of dMMR in MSI-high tumors. These include the *TGF-βRII*, *IGFIIR*, *BAX*, *hMSH6*, and *hMSH3* genes [85,92].

### 2.4. Urothelial Carcinoma

A recent meta-analysis by Chandran et al. showed that dMMR and MSI-H are approximately three times more prevalent in upper tract urothelial cancers (UC) (dMMR 8.95%; MSI-H 8.36%) than in bladder cancer (dMMR 3.09%; MSI-H 2.11%), and that MSI-H occurs more frequently in localized disease compared with metastatic disease [93]. The pattern of MMR protein loss in upper tract UC showed MSH2 and MSH6 to be the most frequent MMR proteins and/or genes lost or altered (both somatic and germline) compared to bladder cancer, the latter for which was unclear. Moreover, a higher risk of developing UC was conferred to those expressing *MSH2* germline mutations compared to individuals harboring germline variants of other MMR genes [94,95]. Several studies have reported that an IHC loss of MSH2 or MSH6 is frequently associated with an underlying germline mutation [39,96,97,98,99]. Furthermore, a majority (64%) of patients with dMMR upper tract UC had previous history of an additional LS-associated neoplasm, most commonly CRC [99]. Similar to the higher frequency evident in endometrial cancers, 12% of cases of MSI-H UC was attributable to the epigenetic silencing of the *MLH1* gene due to promoter hypermethylation. With the exception of a known family history of UC, the National Comprehensive Cancer Network (NCCN) does not recommend routine surveillance for UC in the general population due to a lack of clear supporting evidence. Clinical features of MSI-H UC demonstrate a slightly younger age at presentation (median 60 years) and female predominance [100]. Significant molecular differences were detected in MSI-H UC with higher rate of mutations identified in genes involved in chromatin remodeling and DNA damage response biological pathways [101]. Tumors with dMMR/MSI-H had higher TMB scores compared to MSS tumors, but no difference in programmed death-ligand 1 (PD-L1) expression was evident between the two groups. Distinct histopathological features of MSI-H UC include a papillary histology with an inverted growth pattern, lack of nuclear pleomorphism, presence of pushing borders without destructive infiltrative edges, and increased intratumoral lymphocytes [99]. Prognosis in MSI-H UC is not clearly defined, with most studies demonstrating a favorable prognosis and superior survival compared to MSS UC; however, some studies showed no difference between these two groups [100,102,103,104,105]. Thus, a clear correlation between the MSI-H status and favorable prognosis in UC remains elusive.

### 2.5. Less Common Neoplasms

The dMMR/MSI-H phenotype, while predominantly studied in more common cancers such as CRC and EC, also plays a significant role in other solid tumors, presenting with unique clinical, histopathological, and molecular characteristics. The incidence of MSI-H in rare solid tumors varies but is generally lower compared to that in more commonly recognized malignancies. A high mutational burden, increased lymphocytic infiltration, and a tendency towards poor differentiation are shared features among solid tumors exhibiting the dMMR/MSI-H phenotype. The overall impact of this phenotype on survival and prognosis can vary depending on the tumor type and its specific biological context. Table 2 summarizes various features of less frequently diagnosed MSI-H tumors, the types of which include, but are not limited to, pancreatic cancer, biliary tract cancer, small bowel cancer, prostate adenocarcinoma, ovarian cancer, adrenocortical carcinoma, thyroid cancer, glioblastoma, and non-small cell lung cancer.

## 3. Overview of Therapeutic Approaches in the Treatment of dMMR/MSI-H Solid Tumors

### 3.1. Immunotherapy

Immunotherapy has revolutionized the treatment landscape for dMMR/MSI-H solid tumors, offering significant clinical benefits. The high mutational load in dMMR/MSI-H tumors generates neoantigens which enhances immune recognition through their visibility to the immune system. Additionally, the tumor microenvironment marked by MSI-H disease is notably different than its MSS counterpart, enriched with primed effector T cells that facilitate antitumor response augmented by ICIs. Numerous clinical trials have demonstrated efficacy of ICIs in dMMR/MSI-H tumors, and the survival benefit in metastatic dMMR/MSI-H disease is well established. The status of MSI and MMR in tumors is both prognostic and potentially predictive with respect to outcomes associated with ICI use. The evaluation of dMMR/MSI-H as a biomarker for potential benefit from immunotherapy use has gained importance in treatment decision-making but has not been conclusive in the prediction of response to ICI therapy. Tumor mutational burden has been found to be an independent predictor of benefit for ICI therapy [130]. A possible correlation between TMB and efficacy of ICI treatment was assessed in a large cohort of MSI-H CRC patients; investigators found that patients with MSI-H tumors exhibiting high TMB levels (>37 mut/Mb) were exclusively responders with longer PFS than non-responder patients with low-TMB tumors [130]. As a result, the existence of the hypermutated phenotype exhibited by dMMR/MSI-H tumors further supports the likelihood of an efficacious response to ICI and favorable outcome [128,131,132]. As research is ongoing to determine better predictive biomarkers for ICI response, surrogates have emerged, and data have shown that the immune signature of T and B cells may determine outcomes of ICI therapy [133]. For example, effector T cells showed that T cell repertoire and affinity to neoantigens are highly linked to the effectiveness of ICI therapy [134]. In another study, single-cell RNA sequencing analysis demonstrated that patients with MSI-H CRC who achieved complete pathological response had more CD8^+^ T cells and CD20^+^ B cells in the tumor microenvironment, while those with persistent tumors had more CD4^+^ T regulatory cells [135]. As larger prospective studies incorporate ICI therapy, results from immunologic correlates will continue to add to the growing body of evidence regarding consensus on biomarkers for ICI response.

#### 3.1.1. Immunotherapy Use in Early-Stage Disease

By leveraging the body’s immune system to target cancer cells, perioperative immunotherapy has improved long-term survival when used as part of treatment plans for several solid tumor types. Notably, immune-based treatment of individuals with early stage or localized dMMR/MSI-H CRC, breast cancer, UC and renal cell carcinoma have led to higher response rates as those incurred by their treated counterparts with similarly staged MSS cancers. In individuals with locally advanced dMMR rectal cancer who were treated for 6 months with single-agent dostarlimab (a programmed death receptor-1 [PD-1]-blocking antibody), 100% of treated participants achieved a sustained clinical complete response (CR) (NCT04165772) [136,137]. These results indicate that dostarlimab can effectively induce tumor regression in this specific population and may allow for the avoidance of chemoradiation and surgical resection in this specific setting.

In colon cancer, the NICHE/NICHE-2 (NCT03026140) and NEOPRISM-CRC (NCT05197322) trials provide compelling evidence to support the use of neoadjuvant immunotherapy in MSI-H/dMMR CRC [138,139,140,141]. The NICHE trial reported a 100% pathological response rate, with 95% of patients achieving major pathological responses (≤10% viable tumor cells) and 60% achieving complete pathological responses (no viable tumor cells) [138,139]. Interim analyses from NEOPRISM-CRC mirror these high response rates with 59% of the patients who received neoadjuvant pembrolizumab demonstrating clinical CR rates [140,141]. While the remaining 41% of patients proceeded to surgery, 100% of the patients involved in the trial were cancer-free following treatment and have not experienced disease recurrence months later. 

Immune checkpoint inhibition has also been evaluated in patients who have undergone complete resection of esophageal or gastroesophageal junction (GEJ) cancer subsequent to receiving neoadjuvant chemoradiotherapy, who were determined to have residual pathologic disease [142]. The CheckMate 577 study (NCT02743494) evaluated the efficacy of nivolumab, a PD-1-blocking antibody, in patients with completely resected esophageal or GEJ cancers who had residual pathologic disease following concurrent chemoradiotherapy [142]. A statistically significant improvement in disease-free survival (DFS) was demonstrated for patients receiving nivolumab as compared to those receiving placebo, with this DFS benefit observed regardless of tumor PD-L1 expression and histology. These findings resulted in the U.S Food and Drug Administration (FDA) approval of nivolumab use for the treatment of patients with completely resected esophageal or GEJ cancer with residual pathologic disease who have received neoadjuvant chemoradiotherapy.

Although subgroup analyses of CheckMate 577 did not stratify patients according to MSS or MSI status, the DANTE trial (NCT03421288), which evaluated atezolizumab (a programmed death-ligand 1 [PD-L1] blocking antibody) plus perioperative fluorouracil (5-FU), leucovorin, oxaliplatin, and docetaxel (FLOT) versus FLOT alone in patients with locally advanced, operable adenocarcinoma of the stomach or GEJ, aimed to assess the potential benefit of neoadjuvant chemotherapy with or without ICI in patients with MSI tumors [143]. Investigators found that participants with MSI tumors generally achieved very high rates of pathologic regression following treatment with the combination of atezolizumab and FLOT; among these individuals, rates of pathologic complete or subtotal regression were 80% versus 59% following treatment with chemotherapy without atezolizumab.

Perioperative treatment of patients with locally advanced resectable dMMR/MSI-H gastric/GEJ adenocarcinoma with immunotherapy has been assessed by investigators of the NEONIPIGA trial (NCT04006262) [144]. This single arm phase II study evaluated preoperative nivolumab and ipilimumab and postoperative nivolumab in patients with resectable dMMR/MSI-H gastric/GEJ adenocarcinoma. Pathologic complete response was detected in 59% of 29 patients who underwent surgery, without unexpected immune-related adverse events and/or postoperative morbidity/mortality. Of the 29 patients who underwent resection, 23 received adjuvant nivolumab. Notably, 3 patients were able to forego surgery after confirmed clinical CR was achieved. The potential for organ preservation with this approach is certainly attractive, though further evaluation with larger studies is necessary. After a median follow-up of 14.9 months, there were no relapses to report, although one patient died 3 days after surgery [144].

The IMHOTEP trial (NCT04795661) will be one of the first clinical trials investigating perioperative pembrolizumab in localized resectable dMMR/MSI-H solid tumors in a tumor agnostic setting [145]. With activity already validated in the metastatic setting, it will be interesting to see if the resultant findings reflect a translation into the perioperative curative space and potential FDA approval across tumor types.

#### 3.1.2. Immunotherapy in the Metastatic Setting

Historically, the association of ICI efficacy with dMMR/MSI-H status first emerged with long-term follow up from a multi-institutional, first-in-human, phase I dose-escalation study assessing results anti-PD-1 antibody monotherapy in participants with metastatic treatment–refractory solid tumors (NCT00441337) [146,147]. Three of 39 patients demonstrated objective responses, with one patient with CRC demonstrating a durable CR, with no evidence of disease 3 years following completion of treatment. A second patient with renal cell carcinoma experienced evolution of complete tumor regression off therapy and in remission more than 4 years after discontinuation of anti-PD-1 therapy. The third patient, with melanoma, was found to have a sustained partial response (PR) 16 months following initiation of reinduction therapy with anti-PD-1 therapy. Most notably, as related to the potential impact of dMMR/MSI-H status on response to ICI, however, was the identification of MSI-H in the CRC tumor of the patient achieving durable CR [147]. This phenomenon was later confirmed by Le and co-investigators who showed high response rates and prolonged survival in patients with dMMR/MSI-H tumors treated with PD-1 blockade compared to 0% responses in patients with pMMR tumors [47]. 

Following the launch of numerous subsequent studies evaluating ICIs in a multitude of tumor types in the metastatic setting, a landmark accelerated approval was granted by the FDA in 2017 for the use of pembrolizumab in the treatment of patients with unresectable or metastatic dMMR or MSI-H disease solid tumors that have progressed following prior treatment and who have no satisfactory alternative treatment options, or who specifically have CRC that has progressed following treatment with a fluoropyrimidine, oxaliplatin, and irinotecan. This first-of-its-kind tumor agnostic approval was based on the results of five uncontrolled, open-label, multi-cohort, multi-center, single-arm trials that considered efficacy data evaluated in individuals with MSI-H or dMMR solid tumors treated with pembrolizumab [148]. 

A total of 149 patients with MSI-H or dMMR cancers were identified across these five trials. Ninety-eight percent of these patients had metastatic disease; two percent had locally advanced, unresectable disease. Eighty-four percent of patients with metastatic CRC and 53% of patients with other solid tumors had received two or more prior lines of therapy. Across all five trials, the efficacy analysis for these 149 patients with MSI-H or dMMR disease showed an overall response rate (ORR) of 39.6% (95% CI: 31.7, 47.9) with 7.4% achieving CR and 32.2% exhibiting PR, with 78% of responding patients having responses of 6 months or longer. In patients with CRC, specifically (*n* = 90), the ORR was 36% (95% CI: 26%, 46%) with a duration of response ranging from 1.6+ to 22.7+ months; in patients with other MSI-H or dMMR solid tumors (*n* = 59), the ORR was 46% (95% CI: 33%, 59%) with a duration of response ranging from 1.9+ to 22.1+ months [149]. While the most common tumor types were CRC and EC, other types of malignancies with MSI or dMMR included biliary cancer, GC or GEJ cancer, pancreatic cancer, small intestinal cancer, breast cancer, prostate cancer, bladder cancer, esophageal cancer, sarcoma, thyroid cancer, retroperitoneal adenocarcinoma, small cell lung cancer, and renal cell cancer. Subsequently, data from the KEYNOTE-177 trial (NCT02563002) resulted in the disease-specific FDA approval of ICI for use as first-line therapy for patients with dMMR/MSI-H metastatic CRC, based on the demonstrated superiority of pembrolizumab over standard chemotherapy in this setting, with an ORR observed in 43.8% of the patients in the pembrolizumab group and 33.1% in the chemotherapy group. Among patients with an overall response, 83% in the pembrolizumab group, as compared with 35% of patients in the chemotherapy group, had ongoing responses at 24 months [150]. 

High ICI-induced response rates and improved survival have been seen in the CheckMate 142 trial (NCT02060188), which first explored nivolumab (an anti-PD-1 antibody) monotherapy, and subsequently, nivolumab plus ipilimumab (an anti-CTLA-4 antibody), in previously treated patients with dMMR/MSI-H metastatic CRC [151,152]. In participants receiving nivolumab monotherapy, 31.1% demonstrated an objective response at a median follow-up at 12 months, with 69% having disease control for 12 weeks. At data cut-off, median duration of response had not yet been reached; all responders were alive, and eight had responses lasting 12 months or longer (Kaplan-Meier 12-month estimate 86%, 95% CI 62 to 95) [151]. Among patients who received nivolumab plus ipilimumab, the ORR was 55% (95% CI, 45.2 to 63.8) at a median follow-up of 13.4 months, and the disease control rate for ≥12 weeks was 80%. Median duration of response was not reached; most responses (94%) were ongoing at data cutoff. Progression-free survival rates were 76% (9 months) and 71% (12 months); respective OS rates were 87% and 85% [152]. The results of these studies led to the FDA approval for nivolumab, followed by approval for the addition of ipilimumab to nivolumab therapy, for use in the treatment of patients 12 years of age and older with MSI-H or dMMR metastatic CRC that has progressed following treatment with a fluoropyrimidine, oxaliplatin, and irinotecan.

Overall response rate was 46% (95% CI: 35,58), with 3 CRs and 35 PRs; 89% of responding patients had response durations of ≥6 months. These findings resulted in the FDA approval for the use of this combination of ICIs in the treatment of patients 12 years of age and older with MSI-H or dMMR metastatic CRC that has progressed following treatment with a fluoropyrimidine, oxaliplatin, and irinotecan. The initial CheckMate 142 study evaluating nivolumab and ipilimumab in previously treated individuals with metastatic MSI-H or dMMR CRC further highlighted an observable difference between response rates in LS-CRC vs. sporadic dMMR/MSI-H CRC (ORR 71% vs. 48%, respectively), although these findings must be interpreted while remaining cognizant that LS designation in this case was based on the clinical records of the patients where this reporting was permitted, as genetic testing for LS was not mandated for the protocol [152]. Further, the large number of patients for whom this information was unknown (53 of 119 participants) must be considered.

The evolution of ICI therapy in the treatment of dMMR/MSI-H metastatic disease represents a paradigm shift in oncology, providing durable responses and improved survival for many patients. Although the subset of metastatic solid tumors exhibiting dMMR/MSI-H represents a very small fraction of all metastatic disease, overall, the impact of these molecular features on the potential efficacy of immune-based treatments, in particular, appears to be profound. Given the multitude of approved indications for the use of ICIs (with or without chemotherapy) in the treatment of patients with dMMR/MSI-H metastatic disease in a vast number of tumor types, consideration should be given to tumor MMR/MSI status as a biomarker in algorithms for treatment decision-making.

#### 3.1.3. The Challenge of Resistance to Immunotherapy

Despite the demonstrated success of ICIs in the treatment of individuals with dMMR/MSI-H cancer in various studies, resistance in this population remains a challenge, either as a lack of an initial response (primary resistance) or occurring subsequent to some degree or period of demonstrable clinical benefit (secondary, or acquired, resistance) [153,154]. Both tumor-intrinsic and tumor-extrinsic factors have been identified as influencing sensitivity to immunotherapy, with the intricate interplay between these factors impacting the efficiency of tumor recognition (or “visibility”) and elimination (or “clearance”) by the immune system. Loss of expression of tumor-associated antigens, dysfunctional antigen presentation machinery, aberrant cell signaling pathways, and epigenetic and metabolic alterations may impair T cell function, thus, leading to resistance to immunotherapy. Further, the generation and secretion of factors from cancer cells may modulate the tumor microenvironment, also affecting various immune cell subset activity and function [155].

Similarly, the tumor microenvironment, itself, is comprised of a network of cell populations, cytokines, and metabolites that can influence immune responses. These cell populations, which include myeloid-derived suppressor cells, tumor-associated macrophages, and regulatory T cells, can suppress anti-tumor immune activity, contributing to the resistance to immune-based treatment, particularly immune checkpoint blockade. In addition, host factors, such as the gut microbiota, systemic inflammation, and exposure to previous treatments, may also play roles in immunotherapy resistance [156]. The influence of the gut microbiota, for example, has garnered much attention in recent years, with clinical trials focused on evaluating the impact of microbiota modulation via fecal microbiota transplantation (FMT) on the enhancement of immune checkpoint inhibition in the treatment of individuals with tumors initially shown to be refractory to such treatment (NCT03772899, NCT04758507, NCT05750030, NCT04729322, NCT05251389, and NCT05286294). Results from completed studies support this concept [157,158].

### 3.2. The Impact of dMMR/MSI-H Status on Chemotherapy Efficacy and Outcomes

Several studies suggest that dMMR/MSI-H may be a negative predictive factor for the efficacy of chemotherapy [159]. Chemotherapy resistance seen with MSI-H biology has been most noticeable in the perioperative setting for early-stage disease. Historically, fluoropyrimidine-based chemotherapy appears to be less effective in dMMR/MSI-H CRC compared to pMMR or MSS tumors. In a post hoc analysis of five randomized adjuvant clinical trials, Sargent et al. showed inferior outcomes with 5-FU alone for patients with stage II colon cancer, and no benefit among patients with stage III colon cancer [160]. The addition of oxaliplatin to 5-FU (FOLFOX regimen) appears to improve outcomes in dMMR/MSI-H colorectal cancer to some extent, although the benefit is still less pronounced compared to that evident with pMMR/MSS tumors [161]. In resectable gastric cancer, in a meta-analysis focusing on patients with MSI-H tumors enrolled on the MAGIC (ISRCTN 93793971), CLASSIC (NCT00411229), ARTIST (NCT00323830), or ITACA-S (NCT00323830) trials, the authors confirmed the positive prognostic role of MSI in surgically resected GC and suggest a potential lack of benefit of perioperative or adjuvant chemotherapy for patients with MSI-H GC who have undergone surgery [162].

Combination chemotherapy with ICI in the treatment of dMMR/MSI-H tumors aims at enhancing the efficacy of the treatment by leveraging the synergistic effects of both modalities. Priming of the immune system occurs via the induction of immunogenic cell death, the increased release of tumor antigens, and the enhancement of the immunogenicity of the tumor. In dMMR/MSI-H primary advanced or recurrent endometrial cancer, recent FDA approval for durvalumab in combination with carboplatin and paclitaxel was granted based on results from the DUO-E study (NCT04269200), which demonstrated enrichment in the dMMR patients achieving significant PFS (median PFS not reached) [163].

## 4. Conclusions and Future Perspectives Discussion

The dMMR/MSI-H phenotype represents a distinct subtype posing potentially varied predictive and prognostic implications for solid tumors. While underlying germline mutations in the MMR genes account for a subset of these tumors, the silencing of the *hMLH1* gene through the hypermethylation of the *hMLH1* promoter most likely accounts for the dMMR and MSI observed in tumors that have developed in the sporadic setting. The MSI-H phenotype has been shown to be favorably prognostic for survival, particularly for most surgically resectable solid tumors. The identification of the dMMR/MSI-H status is, thus, crucial for the prognosis and management of various solid tumors, guiding therapeutic decisions and providing valuable insights into the tumor’s biological behavior. The success of immunotherapy, particularly ICIs, and therapeutic regimens utilizing immune-based therapies in combination with or without conventional cytotoxic chemotherapy underscores the potential benefit of immunotherapy in the incorporation of treatment algorithms for this subset of cancers with distinct genetic and molecular features. Ongoing research evaluating resistance mechanisms and the development of novel therapeutic approaches, including strategies focused on the reversal of T cell exhaustion, the reprogramming of the tumor microenvironment to promote an anti-tumor immune response, the increased availability of tumor neoantigens, the modulation of the gut microbiome, the use of small-molecule inhibitors in combination with an immune checkpoint blockade, the utilization of anti-cancer vaccines, and the incorporation of cellular therapies, strive to further enhance the efficacy of immunotherapy, offering hope for even greater advancements in the treatment of dMMR/MSI-H tumors. As solid tumors are increasingly being assessed for dMMR/MSI-H, consensus guidelines would be helpful to facilitate optimal surveillance strategies for affected family members with confirmed germline predisposition.

## Figures and Tables

**Table 1:** Distinctions between MSI-H CRC resulting from germline versus sporadic mutations *.

	Hereditary MSI-H CRC	Sporadic MSI-H CRC
**Clinical Features**	- Associated with early-onset CRC - Typical age < 50 years - Slightly higher male predominance - Predilection for proximal colon, but up to 40% occur in left colon and rectum	- Seen in elderly patients - Typical age > 75 years - Female predominance - Predominantly proximal
**Incidence**	- 2–4% total CRC in the Western world	- 10–15% total CRC
**Histopathology**	- Tubular or tubulovillous adenomas - Crohn’s-like reaction and TILs more common	- Serrated adenomas - Poorly differentiated - Mucinous - Presence of subclones within the tumor (heterogeneity) more common
**Genomics**	- Germline pathogenic variants in DNA MMR genes and *EPCAM* - Up to 50% caused by germline mutation in *hMSH2* - Disruption of the Wnt signaling pathway, with associated *APC* and beta catenin mutations - *KRAS* mutations more common	- Methylation of *hMLH1* promoter due to epigenetic silencing - CIMP characterized by widespread DNA hypermethylation - Concomitant somatic *BRAF* V600E mutation - Reduced frequency to absence of *APC*, *KRAS*, and *TP53* mutations
**Microsatellite Status**	- Lower levels of MSI, with instability in 30–50% of markers	- High levels of MSI with mean positive yield of 87% of markers (range, 50–100%)
**Immunohistochemistry**	- Aberrant beta catenin immunostaining - Concomitant loss of MLH1 and PMS2 - Concomitant loss of MSH2 and MSH6 - Isolated loss of MLH1 (uncommon), PMS2, MSH2, or MSH6	- Virtually all cases associated with loss of expression of hMLH1

* Extrapolated from Young et al. [67] and Jass et al. [68].

**Table 2:** Features of less common tumor types exhibiting dMMR/MSI-H.

	Incidence/Clinical Features	Histopathology	Molecular Features	Prognosis
**Pancreatic ductal cancer**	- 0.8–1.3% of total cases [106] - Predominantly located in the pancreatic body or tail	- Medullary appearance with prominent lymphocytic infiltration [106] - Different histological subtypes such as intraductal papillary mucinous neoplasms and acinar cell carcinoma [106,107] - Associated with poor differentiation	- Associated with germline mutations of *hMLH1* and somatic hypermethylation of the *hMLH1* promoter [108] - Presence of wildtype *KRAS* and *TP53* genes [108] - Frameshift mutations of *hMSH3*, *hMLH3*, *BRCA-2*, *TGF-β type II receptor*, and *BAX* genes evident in MSI-H tumors	- Better prognosis in resectable disease (median survival time: 62 months versus 10 months) [108,109] - Durable responses to anti-PD-1 therapy [110]
**Biliary tract cancer**	- 1–3% of cases [110]	- Associated with mucinous histology [111]	- Associated with a higher TMB value (median TMB 21.7 muts/Mb) and more positive PD-L1 expression [112] - Higher mutation frequencies of *ARID1A*, *ACVR2A*, *TGFBR2*, *KMT2D*, and *RNF43* [112]	- Longer PFS and OS with PD-1 inhibitor-based therapy as compared to those with pMMR tumors receiving same treatment [111] - In patients with resected disease, shorter DFS compared to pMMR patients with resected disease (10.7 months vs. 31.1 months [*p* = 0.025]) [111]
**Small bowel adenocarcinoma**	- 32% of cases [113] - High incidence of metachronous tumors	- IHC loss of MLH1 and MSH2 at similar frequencies [114] - Immune cell infiltration and high PD-1/PD-L1 expression in TILs [113,115]	- High TMB strongly associated with MSI status [115]	- 5-year OS 60% [113] - Lower TNM stage [113,115]
**Prostate adenocarcinoma**	- 3.1% of all cases - 21.9% MSI-H cancers associated with LS [116]	- More likely grade 5 at presentation [117]	- MSH2/*MSH6* most commonly affected [118]	- N1M0 and M1 disease more prevalent [117] - Favorable prognosis; longer PFS [117]
**Ovarian cancer**	- ~2–12% depending on histological type [119]	- Enriched in endometrioid and clear cell histological subtypes [119] - Cribriform, glandular, and mucinous features with predominant tumor-infiltrating lymphocytes; upregulated PD-L1 expression	- *MSH2/MSH6* mutations commonly observed; high TMB [119] - Often associated *with MLH1* promoter hypermethylation in sporadic cases [120] - Frequently co-mutated with *PIK3CA, PTEN,* and *ARID1A* loss	- *Favorable* prognosis, particularly in early stage [119]
**Adrenocortical carcinoma**	- ~4% with MSI-H or dMMR [121]	- Poorly differentiated; presence of necrosis [39] - Often high Weiss score (≥3), consistent with aggressive phenotype [121]	- *MLH1* hypermethylation in sporadic cases; *MSH2/MSH6* loss [121] - Frequent *TP53* alterations	- Overall poor prognosis - Modest benefit from ICIs [121]
**Thyroid cancer**	- ~2–4% in anaplastic forms [122]	- Particularly poorly differentiated or anaplastic [122] - Necrosis and high-mitotic activity in anaplastic forms	- Commonly *MSH2, MLH1*, and *MSH6* mutations; TMB-high profile [122]	- May *respond* to ICIs; otherwise, poor prognosis [122]
**Glioblastoma**	- Approximately 1% of all adult cases [123] - Frontal lobe presentation in more than 50% of cases [123]	- May show giant cell or anaplastic features [124]	- Most frequently lost MMR protein was PMS2 [123]	- Similar recurrence rates in patients, irrespective of MSS or MSI status [123]
**Non-small-cell lung cancer**	- 0.2% to 0.8% of all cases [125] - More prevalent among patients with smoking history [125]	- Mostly squamous cell histology [125]	- Median TMB higher than MSS but no difference in PD-L1 score [125,126] - Compared with MSS patients, MSI-H/dMMR patients had more frame shift mutations [127] - *TP53*, *EGFR*, *LRP1B*, BRCA2, and *NOTCH1* most common mutations in MSI-H lung cancer [126] - MSI-H lung adenocarcinoma with *EGFR* mutations associated with *higher* co-occurring *TGFBR2* and *ERBB2* mutation rates [126]	- High response rates to ICIs [128] - Improved OS and PFS with ICIs, though dependent on comorbidities and stage [129]

TNM = tumor node metastases; mut/Mb = mutations/Megabase.

## Data Availability

Not applicable.

## Abbreviations

The following abbreviations are used in this manuscript:

5-FU	5-fluorouracil
BER	Base excision repair
CIMP	CpG island methylator phenotype
CIN	Chromosomal instability
CRC	Colorectal cancer
DFS	Disease-free survival
dMMR	Deficient mismatch repair
EC	Endometrial cancer
EPCAM	Epithelial cellular adhesion molecule
FDA	Food and Drug Administration
FLOT	Fluorouracil, leucovorin, oxaliplatin, and docetaxel
FMT	Fecal microbiota transplantation
FOLFOX	Folinic acid (leucovorin), fluorouracil, and oxaliplatin
GC	Gastric cancer
GEJ	Gastroesophageal junction
HLA	Human leukocyte antigen
HNPCC	Hereditary non-polyposis
ICI	Immune checkpoint inhibitor
IDLs	Insertion–deletion loops
IHC	Immunohistochemistry
LOH	Loss of heterozygosity
LS	Lynch syndrome
LS-CRC	Lynch syndrome-associated colorectal cancer
LVI	Lymphovascular invasion
MAP M	UTYH-associated polyposis
MMR	Mismatch repair
MSI	Microsatellite instability
MSI-H	Microsatellite instability high
MSS	Microsatellite stable/microsatellite stability
NCCN	National Comprehensive Cancer Network
OS	Overall survival
ORR	Overall response rate
PCR	Polymerase chain reaction
PD-1	Programmed cell death protein 1
PD-L1	Programmed death-ligand 1
PFS	Progression-free survival
pMMR	Proficient mismatch repair
PPAP	Polymerase proofreading-associated polyposis
TCGA	The Cancer Genome Atlas
TILs	Tumor-infiltrating lymphocytes
TMB	Tumor mutational burden
UC	Urothelial cancer
